# Selenium Lessens Osteoarthritis by Protecting Articular Chondrocytes from Oxidative Damage through Nrf2 and NF-κB Pathways

**DOI:** 10.3390/ijms25052511

**Published:** 2024-02-21

**Authors:** Hsiao-Ling Cheng, Chia-Chi Yen, Li-Wen Huang, Yu-Chen Hu, Tzu-Ching Huang, Bau-Shan Hsieh, Kee-Lung Chang

**Affiliations:** 1 Department of Pharmacy, Kaohsiung Municipal Min-Sheng Hospital, Kaohsiung 802511, Taiwan; chenghl.tanya@gmail.com; 2 Department of Orthopedics, Kaohsiung Municipal Min-Sheng Hospital, Kaohsiung 802511, Taiwan; ycc.lun@msa.hinet.net; 3 Department of Medical Laboratory Science and Biotechnology, College of Health Sciences, Kaohsiung Medical University, Kaohsiung 807378, Taiwan; lewehu@cc.kmu.edu.tw; 4 Department of Biochemistry, School of Medicine, College of Medicine, Kaohsiung Medical University, Kaohsiung 807378, Taiwan; chingshouhu@gmail.com (Y.-C.H.); huangtavia@gmail.com (T.-C.H.); 5 Graduate Institute of Medicine, College of Medicine, Kaohsiung Medical University, Kaohsiung 807378, Taiwan; hsiehbs@gmail.com

**Keywords:** selenium, osteoarthritis, chondrocyte, antioxidant, anti-inflammation, minerals

## Abstract

Osteoarthritis (OA) causes joint pain and disability due to the abnormal production of inflammatory cytokines and reactive oxygen species (ROS) in chondrocytes, leading to cell death and cartilage matrix destruction. Selenium (Se) intake can protect cells against oxidative damage. It is still unknown whether Se supplementation is beneficial for OA. This study investigated the effects of Se on sodium iodoacetate (MIA)-imitated OA progress in human chondrocyte cell line (SW1353 cells) and rats. The results showed that 0.3 μM of Se treatment could protect SW1353 cells from MIA-induced damage by the Nrf2 pathway by promoting the gene expression of glutathione-synthesis-related enzymes such as the glutamate–cysteine ligase catalytic subunit, the glutamate–cysteine ligase modifier subunit, and glutathione synthetase. In addition, glutathione, superoxide dismutase, glutathione peroxidase, and glutathione reductase expressions are also elevated to eliminate excessive ROS production. Moreover, Se could downregulate NF-κB, leading to a decrease in cytokines, matrix proteases, and glycosaminoglycans. In the rats, MIA-induced cartilage loss was lessened after 2 weeks of Se supplementation by oral gavage; meanwhile, glutathione synthesis was increased, and the expressions of pro-inflammatory cytokines were decreased. These results suggest that Se intake is beneficial for OA due to its effects of decreasing cartilage loss by enhancing antioxidant capacity and reducing inflammation.

## 1. Introduction

Osteoarthritis (OA) is the most common degenerative arthropathy among the elderly, affecting around 10% of men and 18% of women over 60 years of age [1,2]. The aging-caused changes in the status of oxidative stress and inflammation have been recognized as important factors in OA pathology [3,4]. Chondrocytes are the solitary cell type found in cartilage and participate in the degeneration process of OA [5]. In OA progressions, chondrocytes may produce excessive amounts of reactive oxygen species (ROS); simultaneously, this triggers the activation of the nuclear factor kappa B (NF-κB) signaling pathways [6]. This activation may cause an increase in inflammatory cytokines like TNF-α, IL-1β, and IL-6, as well as an increase in the expression of matrix metalloproteinases (MMPs) (i.e., MMP-1,-3,-9,-13) [5]. In addition, there is an increase in disintegrins and metalloproteinases with increasing thrombospondin motifs (ADAMTSs) (i.e., ADAMTS-4) [5]. There are two primary extracellular matrix proteins present in cartilage: type II collagen and aggrecan [7]. During the pathological physiology of cartilage, MMPs play roles in the breakdown of collagen, while ADAMTSs play roles in the breakdown of the proteoglycan molecule aggrecan. Accordingly, these two enzymes are related to the destruction of the matrix [8].

Previous studies have indicated that Se deficiency could cause cell damage, leading to diseases, including OA [9] and Kashin–Beck disease (KBD) [10], in humans through multifaceted mechanisms. One report showed that Se supplementation helps reduce the development of mechanically induced osteoarthritis in the knee joints of STR/1N mice [11]. As is well known, increased oxidative stress and inflammation in chondrocytes are the main causes of OA progression; meanwhile, Se has antioxidative and anti-inflammatory effects. In addition, almost all genes related to antioxidative factors, such as the glutamate–cysteine ligase catalytic subunit (GCLC), the glutamate–cysteine ligase modifier subunit (GCLM), glutathione peroxidase (GPx), and superoxide dismutase (SOD), are transcriptionally regulated by nuclear factor E2-related factor 2 (Nrf2) [12,13]. As a result, Nrf2 can reduce intracellular ROS levels and thus inhibit the NF-κB signaling pathway, which leads to a decrease in the expression of pro-inflammatory cytokines such as IL-1, IL-6, and TNF-α [14,15]. Similarly, previous studies have reported that the Se-activating Nrf2 pathway can protect cells from damage [16,17]. Therefore, it is rational to propose that Se supplementation would be beneficial for OA treatment. Herein, we investigate the effects and underlying mechanisms of sodium selenite (Na_2_SeO_3,_ Se) on MIA-imitated OA progress in human chondrocyte cell line (SW1353 cells) and in Wistar rats.

## 2. Results

### 2.1. Cell Viability

**Figure 1:**
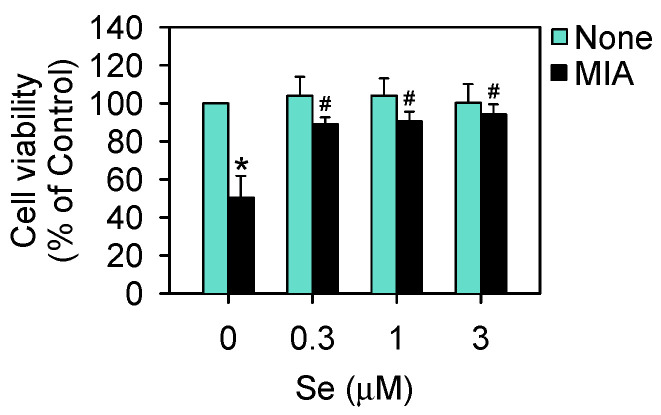
Cell viability of SW1353 cells treated with MIA and/or Se. SW1353 cells were cultured for 24 h with or without 5 μM of MIA, accompanied by the presence or absence of 0.3–3 μM of Se. Subsequently, cell viability was assessed using trypan blue exclusion and represented as a percentage relative to the control. The results are presented as the mean ± standard deviation of experiments performed in triplicate. *: *p* < 0.05 versus untreated control group; #: *p* < 0.05 versus MIA-treated group.

MIA is an inhibitor of glyceraldehyde-3-phosphate dehydrogenase (GAPDH) and can induce chondrocyte death. Our previous finding demonstrated that treatment with 5 μM of MIA could induce SW1353 cell damage or even death over different treatment periods [18,19]. Accordingly, a dose of 5 μM of MIA was recognized as optimal for mimicking OA progression in humans. To determine whether additional Se treatment may affect cell viability or not, the cells were incubated with or without 5 μM of MIA and in the presence of 0–3 μM of Se for 24 h, and then cell viability was analyzed. As shown in Figure 1, cell viability was not significantly altered by Se in the groups without MIA treatment, even at a concentration of 3 μM of Se. In contrast, cell viability was markedly decreased in the MIA-treated group, whereas the addition of Se could prevent a decrease in cell viability. Notably, a concentration of 0.3 μM of Se could reach the same efficacy as 3 μM of Se. Therefore, we selected concentrations of 5 μM of MIA and 0.3 μM of Se for the following cell culture experiments.

### 2.2. Oxidative Stress and Antioxidants

**Figure 2:**
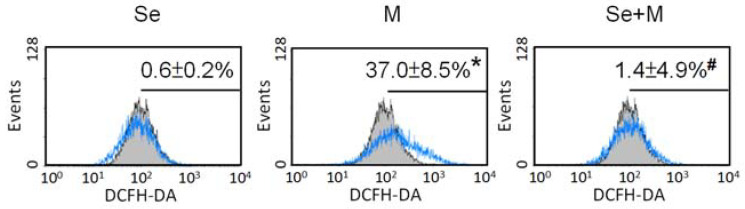
Oxidative stress of SW1353 cells treated with MIA (M) and/or Se. SW1353 cells were cultured for 24 h with or without 5 μM of MIA, accompanied by the presence or absence of 0.3 μM of Se. Subsequently, levels of ROS were assessed using 10 μM of DCFH-DA staining, followed by analysis through flow cytometry. The untreated control is depicted by the gray-filled area with black lines, while the treated groups are represented by the blue lines. The results are presented as the mean ± standard deviation of experiments performed in triplicate. *: *p* < 0.05 versus untreated control group; #: *p* < 0.05 versus MIA-treated group.

**Figure 3:**
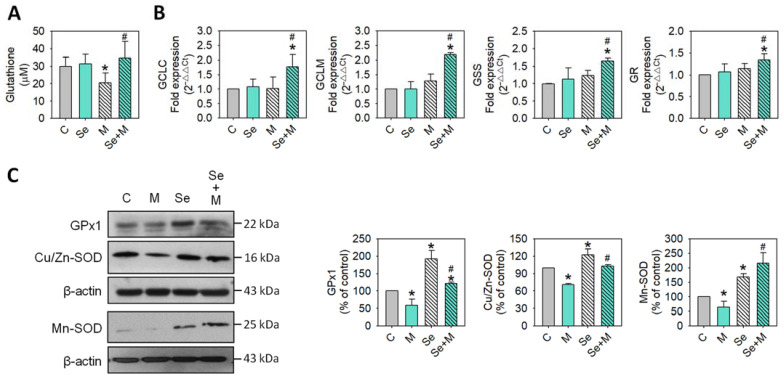
Expression of antioxidants in SW1353 cells treated with MIA and/or Se. SW1353 cells were cultured for 24 h with or without 5 μM of MIA, accompanied by the presence or absence of 0.3 μM of Se. (**A**) The GSH levels were assayed by the enzymatic recycling method. (**B**) The mRNA expressions of GCLC, GCLM, GSS, and GR were evaluated by RT-qPCR and expressed as fold changes relative to the untreated control group. (**C**) The protein levels of GPx1, Cu/Zn-SOD, and Mn-SOD were determined using Western blotting, with β-actin used as a loading control. The right panel shows the relative density values of the representative Western blots compared to the untreated control (100%). The results are presented as the mean ± standard deviation of experiments performed in triplicate. *: *p* < 0.05 versus untreated control group; #: *p* < 0.05 versus MIA-treated group.

The status of oxidative stress was evaluated by ROS production, and SW1353 cells were treated with or without 5 μM of MIA and in the presence or absence of 0.3 μM of Se for 24 h. As shown in Figure 2, MIA could induce ROS production, while Se addition decreased ROS production. In addition, the antioxidative status was analyzed through the detection of glutathione (GSH) levels and related GSH synthesis enzymes. The GSH levels were decreased by MIA, whereas Se addition might have diminished this decrease (Figure 3A). Moreover, the mRNA expression of the GSH-synthesis-related key enzymes GCLC, GCLM, glutathione synthetase (GSS), and glutathione reductase (GR) increased after Se addition (Figure 3B), suggesting that an increase in GCLC, GCLM, GSS, and GR expression contributes to increased GSH levels.

**Figure 4:**
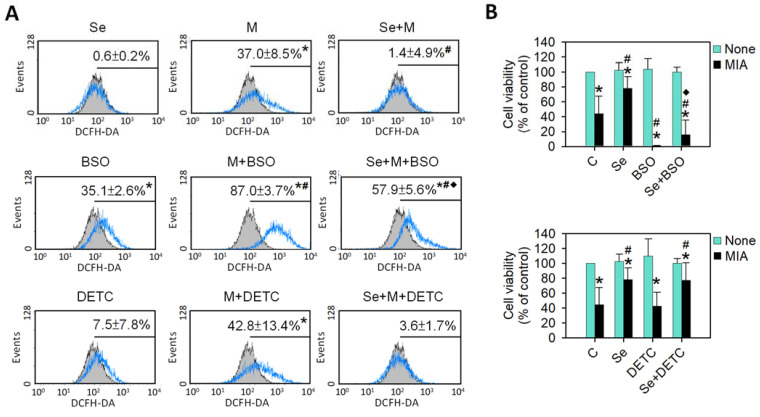
Effects of an inhibitor of GSH or SOD on the intracellular ROS levels and cell viability in SW1353 cells. SW1353 cells were subjected to treatment with or without 5 μM of MIA and in the presence or absence of 0.3 μM of Se, with the addition of 10 μM of BSO (a GSH inhibitor) or 0.2 μM of DETC (a SOD inhibitor) for 24 h. (**A**) Intracellular levels of ROS were assessed through 10 μM of DCFH-DA staining, followed by analysis using flow cytometry. The untreated control is depicted by the gray-filled area with black lines, while the treated groups are represented by the blue lines. (**B**) The cell viability was evaluated using trypan blue exclusion and expressed as a percentage of the control. The results are presented as the mean ± standard deviation of experiments performed in triplicate. *: *p* < 0.05 versus untreated control group; #: *p* < 0.05 versus MIA-treated group; ◆: *p* < 0.05 versus MIA+Se-treated group.

At the same time, the antioxidative enzyme expression of GPx1, Cu/Zn-SOD, and Mn-SOD was also analyzed. Figure 3C shows that Se alone could significantly increase GPx1, Cu/Zn-SOD, and Mn-SOD expression, whereas MIA decreased GPx1, Cu/Zn-SOD, and Mn-SOD expression. In the cotreatment with MIA and Se, the Se was able to prevent the MIA-induced decrease in GPx1, Cu/Zn-SOD, and Mn-SOD. Furthermore, to clarify the major antioxidative role in Se protection, an inhibitor of GSH or SOD was added to the MIA/Se-cotreated cells, and then ROS and cell viability were analyzed. As shown in Figure 4, the addition of buthionine sulfoximine (BSO), a GSH inhibitor, abated the effects of Se, but sodium diethyldithiocarbamate (DETC), a SOD inhibitor, did not affect it. From this, it can be inferred that GSH is more effective than SOD in the protection of Se. 

### 2.3. Matrix Synthesis and Degradation

**Figure 5:**
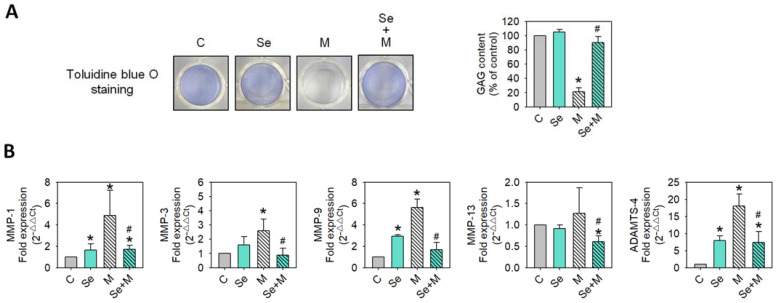
Extracellular matrix and MMP expressions of SW1353 cells treated with MIA and/or Se. SW1353 cells were cultured for 24 h with or without 5 μM of MIA, accompanied by the presence or absence of 0.3 μM of Se. (**A**) Glycosaminoglycan (GAG) contents were assessed by toluidine blue O staining, followed by a colorimetric assay. (**B**) The mRNA expressions of MMP-1, MMP-3, MMP-9, MMP-13, and ADAMTS-4 were evaluated by RT-qPCR and expressed as fold changes relative to the untreated control group. The results are presented as the mean ± standard deviation of experiments performed in triplicate. *: *p* < 0.05 versus untreated control group; #: *p* < 0.05 versus MIA-treated group.

To determine whether extracellular matrix synthesis and degradation were affected by MIA and/or Se, the SW1353 cells were treated with/without 5 μM of MIA and/or 0.3 μM of Se for 24 h, and then acidic polysaccharide contents were examined by toluidine blue O staining, and MMP-1, MMP-3, MMP-9, MMP-13, and ADAMTS-4 expressions were analyzed by RT-qPCR. The results showed that MIA significantly decreased acidic polysaccharide levels (Figure 5A), while Se addition was able to significantly inhibit this decrease during matrix synthesis. Figure 5B shows that MIA dramatically enhanced the expressions of MMP-1, MMP-3, MMP-9, and ADAMTS-4, while Se addition was able to diminish these increases, suggesting that Se may lessen the MIA-induced matrix loss.

### 2.4. Se Reduces MIA-Induced Inflammatory Cytokine Production

**Figure 6:**
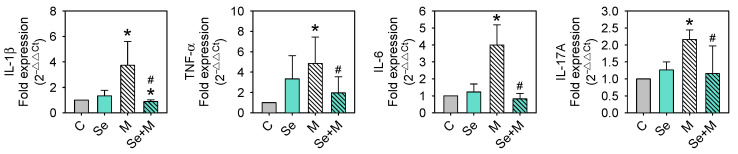
Inflammatory cytokine expressions of SW1353 cells treated with MIA and/or Se. SW1353 cells were cultured for 24 h with or without 5 μM of MIA, accompanied by the presence or absence of 0.3 μM of Se. The mRNA levels of IL-1β, TNF-α, IL-6, and IL-17A were evaluated through RT-qPCR and expressed as fold changes relative to the untreated control group. The results are presented as the mean ± standard deviation of experiments performed in triplicate. *: *p* < 0.05 versus untreated control group; #: *p* < 0.05 versus MIA-treated group.

To examine whether proinflammatory cytokines involved in OA were affected, the mRNA expressions of IL-1β, TNF-α, IL-6, and IL-17A were assayed using RT-qPCR. As shown in Figure 6 the MIA treatment increased IL-1β, TNF-α, IL-6, and IL-17A mRNA expression, and the addition of Se to MIA-treated cells augmented these increases. 

### 2.5. Nrf2 and NF-κB Activation

**Figure 7:**
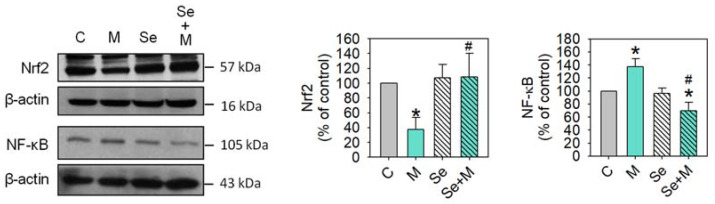
Transcription factor expressions of SW1353 cells treated with MIA and/or Se. SW1353 cells were cultured for 24 h with or without 5 μM of MIA, accompanied by the presence or absence of 0.3 μM of Se. The protein levels of Nrf2 and NF-κB were determined using Western blotting, with β-actin used as a loading control. The right panel shows the relative density values of the representative Western blots compared to the untreated control (100%). The results are presented as the mean ± standard deviation of experiments performed in triplicate. *: *p* < 0.05 versus untreated control group; #: *p* < 0.05 versus MIA-treated group.

Nrf2 is a transcription factor that is crucial for cellular defense against oxidative stress by regulating genes involved in antioxidative responses [20]. On the other hand, certain inflammatory mediators, such as IL-1β, TNF-α, IL-6, and IL-17A, can activate another pathway called the NF-κB pathway, which leads to the production of collagenases and aggrecanases (including MMP-1, MMP-3, MMP-13, and ADAMTS-4), resulting in the breaking down of the extracellular matrix and leading to degradation [21]. This may lead to a positive feedback loop; therefore, more inflammatory mediators are created, which may worsen the situation [22,23]. This study found that MIA treatment caused changes in oxidative stress, antioxidative enzyme expression, and inflammatory cytokines. Moreover, Se addition was able to decrease these MIA-induced changes. Then, we investigated whether these changes were linked to the expression of Nrf2 and/or NF-κB. Figure 7 shows that MIA dramatically decreases Nrf2 expression, and Se can prevent this MIA-induced decrease in Nrf2 expression. In contrast, MIA increases NF-κB expression, and Se not only prevents this increase but also decreases NF-κB expression to values even lower than the control levels.

### 2.6. Se Supplementation Lessens OA Progression in Rats

**Figure 8:**
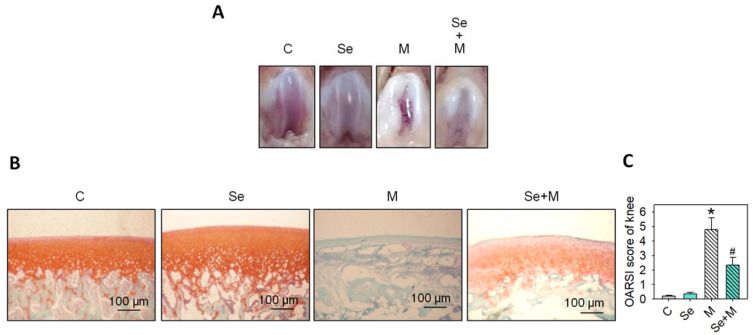
Morphological and histological changes in articular cartilage in the MIA-induced OA rats with or without Se supplementation. The study design and timeline for the experiment involving MIA-induced OA in rats and Se administration are outlined in the Materials and Methods section. (**A**) Pictures capturing the articular surfaces of the femoral groove. (**B**) Photomicrographs depicting histomorphological alterations in joint cartilage stained with safranin O/fast green. Proteoglycan is represented by the red color. Scale bar: 100 μm. (**C**) Scoring of each joint based on the OARSI criteria. The results are presented as the mean ± standard deviation (*n* = 6). *: *p* < 0.05 versus untreated control group; #: *p* < 0.05 versus MIA-treated group.

To investigate whether that found in vitro (SW1353 cells) was similar to that in vivo rat with MIA-induced osteoarthritis (OA) progression, the rats receiving Se supplementation for two weeks had a cartilage morphology similar to that of the control group. In contrast, the group without Se supplementation showed severe erosion, synovial hypertrophy, and cartilage defects, indicating significant arthritic progression (as seen in Figure 8A). This indicates that Se supplementation can mitigate the arthritic progression in the MIA-treated rats. A histopathological examination of the rats’ left joints was conducted using safranin O and fast green stains, followed by semi-quantitative Osteoarthritis Research Society International (OARSI) scoring to assess the degree of joint cartilage matrix loss and recovery, where a higher score indicates more severe joint damage. The MIA-treated groups showed higher OARSI scores with a lack of safranin O staining (red color) and positive fast green staining, suggesting the absence of acidic proteoglycan in the cartilage. Conversely, the MIA-treated groups supplemented with Se showed lower OARSI scores and positive safranin O staining, indicating that the Se supplementation was able to mitigate proteoglycan loss in the cartilage (Figure 8B,C). Supplementary Figure S1 displays additional tissue section images of cartilage.

**Figure 9:**
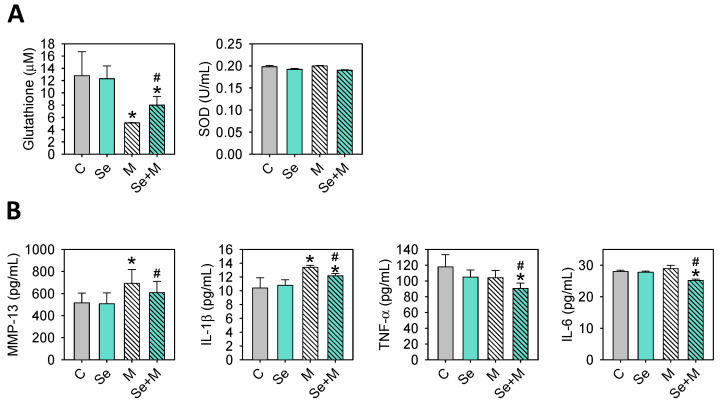
Serum levels of GSH, SOD, MMP-13, and cytokines in the MIA-induced OA rats with or without Se supplementation. (**A**) GSH and SOD levels were assessed using enzymatic and colorimetric assay kits. (**B**) MMP-13, IL-1β, TNF-α, and IL-6 levels were determined through enzyme-linked immunosorbent assay. The results are presented as the mean ± standard deviation (*n* = 6). *: *p* < 0.05 compared to the untreated control group; #: *p* < 0.05 compared to the MIA-treated group.

Simultaneously, the antioxidative and anti-inflammatory capacity, as well as the levels of GSH, SOD, MMP-13, IL-1β, TNF-α, and IL-6 in serum, were evaluated. The results showed (Figure 9) that Se supplementation in the MIA-treated rats led to higher GSH levels and lower MMP-13, IL-1β, TNF-α, and IL-6 levels as compared with those without Se supplementation.

## 3. Discussion

**Figure 10:**
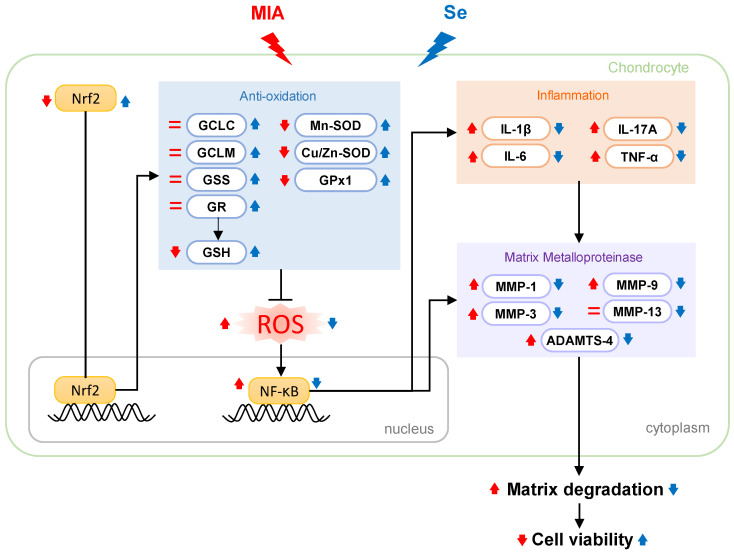
Schematic diagram illustrating the effects of Se on MIA-treated chondrocytes. The findings of this study highlight that Se can protect against MIA-induced oxidative stress, elevated levels of pro-inflammatory cytokines (IL-1β, IL-6, IL-17A, and TNF-α), and increased expression of MMPs (MMP-1, MMP-3, MMP-9, MMP-13, and ADAMTS-4). This protection is attributed to the activation of the Nrf2 pathway, which, in turn, upregulates the gene expression of antioxidants, including Cu/Zn-SOD, Mn-SOD, GPx1, GSH, GCLC, GCLM, GSS, and GR. This cascade leads to an augmentation of the anti-oxidative capacity, providing defense against MIA-induced oxidative stress. Furthermore, the addition of Se results in a decrease in NF-κB expression, counteracting the increase induced by MIA. This decrease contributes to a reduction in pro-inflammatory cytokines and MMP expression, ultimately mitigating matrix degradation. Red↑: enhanced by MIA; Red↓: decreased by MIA; Red=: did not change with MIA; Blue↑: enhanced by Se; Blue↓: decreased by Se.

Overall, these results demonstrate that Se can decrease MIA-induced damage to chondrocytes and OA progress in rats with MIA treatment. Herein, the results of the cell culture showed that 0.3 μM of Se treatment could protect SW1353 cells from MIA-induced damage through the activation of the Nrf2 pathway to promote the gene expression of GSH-synthesis-related enzymes, including GCLC, GCLM, and GSS. Additionally, in the co-addition of MIA and Se group, the GSH levels and the expressions of the antioxidation-related enzymes, SOD, GPx1, and GR were increased, which eliminated the MIA-induced excessive ROS production. Furthermore, Se can downregulate NF-κB, resulting in a decrease in the inflammatory cytokines IL-1β, TNF-α, IL-6, and IL-17A. Similarly, the matrix proteases of MMP-1, MMP-3, MMP-9, MMP-13, and ADAMTS-4 were decreased through Se addition to the MIA-treated group (Figure 10). These findings from the cell culture are almost all obtained in the MIA-induced OA rats model, including the enhancement of the capacities of antioxidative and inflammatory defenses through Se supplementation.

Although Se has antioxidant and anti-inflammatory properties, an excessive intake of Se may cause selenosis [13], which should be taken into consideration. Accordingly, it is important to search for a safe dosage of Se supplementation. The recommended daily intake of Se for adult men and women is 55 μg, with an upper limit of 400 μg [24]. The average Se content in human blood is 100 μg/L (1.27 μM) [25], and toxicity occurs at concentrations exceeding 400 μg/L (5.06 μM) [26]. Currently, there are Se-related health foods available on the market which recommend a daily intake of 200 μg or 3.3 μg/kg for adults weighing 60 kg. To convert this dosage for rats, we multiply it by 6.2 as per the body surface area (BSA) dose conversion equation [27]. This results in a dosage of 20.7 μg/kg for rats. Therefore, in our research, we used Se at a concentration of 0.3–3 μM and administered a dose of 20.7 μg/kg in the human chondrosarcoma cell line SW1353 and in rats. Our findings revealed that Se at a concentration of 0.3–3 μM is a safe dose and can protect the human chondroma cell line SW1353 from damage caused by MIA. Furthermore, rats fed with Se at a dose of 20.7 μg/kg for two weeks showed a decrease in damage caused by MIA-induced degenerative arthritis. Based on epidemiological data from China, it is suggested that a daily exposure of 750–850 μg is the upper limit of safety [28]. A recent small intervention trial involving doses of 1600 and 3200 μg/day showed no signs of serious toxicity with relatively long-term supplementation [29]. Based on the above findings and reports, the recommendation of daily 200 μg Se supplementation for adults weighing 60 kg (3.3 μg/kg) is in a safe range and can effectively protect patients from OA progression without harming their health.

Se has a pivotal role in maintaining human health, mainly due to its anti-oxidation, anti-inflammation, chemo-prevention, and antiviral properties related to improving immune responses [30]. Based on our findings, Se can stimulate chondrocytes to produce antioxidants like SOD, GSH, GPx1, and GR through the regulation of the Nrf2 pathway, through which oxidative stress can be reduced within cells. In another way, Se can decrease NF-κB expression to relieve inflammation and slow down cartilage matrix loss.

Nrf2 is a transcription factor that plays an important role in phase II antioxidant enzyme expression and cellular defense against oxidative stress. It tightly regulates GSH levels by directly controlling the expression of the two subunits that constitute the glutamate–cysteine ligase (GCL) complex: the catalytic subunit (GCLC) and the modifier subunit (GCLM) [31]. GCL catalyzes the reaction of glutamate with cysteine, the rate-limiting step in the synthesis of GSH [32]. Reduced glutathione (GSH) is considered one of the most important scavengers of ROS, and oxidized glutathione (GSSG) is regarded as a marker of oxidative stress. When GSH is depleted, chondrocytes are more susceptible to oxidant-mediated cell death [33,34]. Our results also showed that the GSH synthesis inhibitor BSO synergistically enhanced MIA-induced SW1353 cell death, and it can diminish the protection of Se, but that is not found in the case of DETC, a SOD inhibitor. These findings suggest that GSH plays a more important role than SOD in the protection of Se against oxidative stress. 

Nrf2 not only regulates intracellular antioxidant activity but also regulates anti-inflammatory effects. One report showed that the activity of NF-κB and the expression of inflammatory cytokines, including TNF-α, IL-1β, IL-6, and MMP-9, were more obviously upregulated in Nrf2 knockout astrocytes [35]. These results suggest that a loss of Nrf2 may lead to aggressive inflammation by activating NF-κB and increasing downstream pro-inflammatory cytokine expression. However, NF-κB–DNA binding activity was significantly inhibited in a diabetic mouse model with Nrf2 overexpression [36], which suggests the negative regulation of NF-κB by Nrf2. Our study has similar results: Se increases Nrf2 expression but decreases NF-κB expression, leading to the prevention of MIA-induced increases in inflammation and matrix degradation. In particular, under normal circumstances, the levels of Nrf2 are maintained at a low level due to its constant binding to Keap1, which leads to its degradation via the proteasome. However, when a cell is exposed to stressors such as inflammation, oxidative stress, or changes in metabolism, the levels of Nrf2 can temporarily or continuously increase due to disruption of the Keap1–Nrf2 complex, resulting in the activation of Nrf2, and Nrf2 moves to the nucleus, where it interacts with various transcription factors and cofactors to regulate antioxidant defense, anti-inflammation, and detoxification processes [20]. Currently, there are at least three known ways for Nrf2 to inhibit the NF-κB pathway [37]: (1) Keap1 acts as an E3 ubiquitin ligase for IκB kinase β (IKKβ). It can phosphorylate the inhibitor I-κB, allowing for the downregulation of the NF-κB signaling pathway [38]; (2) the Rho GTPase (RAC1) protein activates NF-κB transcriptional levels, leading to an upregulation of Nrf2 expression, which then inhibits NF-κB in a regulatory feedback loop [39]; and (3) Nrf2 can block the nuclear translocation of the NF-κB pathway by regulating the downstream antioxidant gene HO-1, thereby inhibiting the inflammatory response induced by NF-κB [40].

There are several limitations to the present study since the experimental results of the cell culture and rat model are not fully applicable to humans. Therefore, we were unable to determine if the findings of this study are applicable to humans. In particular, the optimal Se dosage with maximal efficacy and no toxicity for patients as protection against OA progression still needs further study through a clinical trial. Similarly, we also cannot be sure if these effects of Se are reversible after Se withdrawal, as our design did not include a withdrawal period. Furthermore, since nuclear and cytosolic fractions were not separated in the Western blotting of the Nrf2 analysis, we are not sure whether nuclear translocation contributes to the change in Nrf2 signaling by Se or not. In other words, this study only assessed the increase in Nrf2 activation but not how it occurred.

Taken together, our results demonstrate that Se might protect chondrocytes from MIA-induced cell damage and cartilage loss by increasing antioxidant capacity through the Nrf2 pathway and inhibiting inflammation through the NF-κB pathway, suggesting that Se supplementation may be beneficial to humans in the treatment of OA progression. The present study provides a new direction for Se supplementation in addition to the traditional use of glucosamine sulfate and chondroitin sulfate for joint health care. 

## 4. Materials and Methods

### 4.1. Cell Culture and Chemicals

The chondrosarcoma cell line, SW1353, derived from a human source, was acquired from the Bioresource Collection and Research Center (BCRC) at the Food Industry Research and Development Institute in Hsinchu, Taiwan. The cells were cultivated using Dulbecco’s modified Eagle medium (DMEM) (Gibco BRL, Grand Island, NY, USA) supplemented with 100 units/mL of penicillin, 100 μg/mL streptomycin (Gibco BRL, Grand Island, NY, USA), and 10% fetal bovine serum (FBS) (HyClone, Auckland, NZ, USA) in a 5% CO_2_ incubator at 37 °C. The cells were seeded at a density of 5 × 10^5^ onto 6 cm dishes in DMEM, allowing for attachment over 16 h. Subsequently, the cells were treated with varying concentrations of selenium (Se) in the presence or absence of 5 μM of MIA for a specified duration, followed by an analysis of the observed effects. MIA, Se, and all other drugs and chemicals of analytical grade were procured from Sigma-Aldrich Co., LLC (St. Louis, MO, USA).

### 4.2. Cell Viability Assay

SW1353 cells were seeded at a density of 8 × 10^4^ cells per well in 24-well plates. The cells were then treated with 5 μM of MIA and varying concentrations of Se (ranging from 0 to 3 μM) for 24 h. Subsequently, the cells were harvested. Viable cell counts were determined using a dye exclusion technique employing 0.4% trypan blue (Gibco BRL, Grand Island, NY, USA) and counted with a hemocytometer. All cell counts were carried out in triplicate for accuracy and consistency.

### 4.3. Cell Viability in the Presence of a GSH or SOD Inhibitor

SW1353 cells were seeded at a density of 8 × 10^4^ cells per well in 24-well plates. Before treatment, the cells were subjected to a 1 h pretreatment with either 10 μM of buthionine sulfoximine (BSO) (Sigma-Aldrich Co., LLC, St. Louis, MO, USA), a GSH inhibitor, or 0.2 μM of diethyldithiocarbamate (DETC) (Sigma-Aldrich Co., LLC, St. Louis, MO, USA), a SOD inhibitor. Following the pretreatment, the cells were exposed to 5 μM of MIA and/or 0.3 μM of Se for 24 h, after which cell viability was assessed. All cell viability measurements were carried out in triplicate for accuracy and consistency.

### 4.4. Measurement of ROS

SW1353 cells were seeded at a density of 4 × 10^5^ cells in 6 cm dishes. The cells were then treated with MIA and/or Se for 24 h. Subsequently, intracellular reactive oxygen species (ROS) were assessed using 10 μM of 2′,7′-dichlorofluorescein diacetate (DCFH-DA) (Molecular Probes, Eugene, OR, USA), following the protocol outlined in our prior study [41]. Each experiment was carried out in triplicate for accuracy and consistency.

### 4.5. Measurement of GSH

SW1353 cells were seeded at a density of 4 × 10^5^ cells in 6 cm dishes. The cells were then treated with MIA and/or Se for 24 h. After treatment, intracellular GSH levels were quantified using a GSH assay kit (Item No. 703002) (Cayman Chemical, Ann Arbor, MI, USA), following the manufacturer’s instructions. Each experiment was carried out in triplicate for accuracy and consistency.

### 4.6. Toluidine Blue O Staining

SW1353 cells were seeded at a density of 4 × 10^5^ cells in 6 cm dishes. The cells were then treated with MIA and/or Se for 24 h. Following treatment, the culture medium was removed, and the cells were washed with phosphate-buffered saline (PBS). Subsequently, the cells were fixed using 4% paraformaldehyde in PBS for 10 min at room temperature (RT) and then rinsed with PBS. The fixed cells were then incubated with a 0.5% toluidine blue O staining solution for 30 min at RT. After washing with PBS, images of the staining were captured using a digital camera. To quantify the toluidine blue O pigment, it was extracted by incubating the cells with 6 mol/L guanidine-HCl (Sigma-Aldrich Co., LLC, St. Louis, MO, USA) for 10 min at RT. The absorbance of the extracts was measured at 630 nm using a microplate reader (BioTek Synergy H1 Plate Reader, BioTek Instruments Inc., Winooski, VT, USA). This procedure was performed to assess and quantify the staining intensity. Each experiment was carried out in triplicate for accuracy and consistency.

### 4.7. Western Blot Analysis

SW1353 cells were seeded at a density of 4 × 10^5^ cells in 6 cm dishes. The cells were then treated with MIA and/or Se for 24 h. After treatment, cell extracts were prepared for Western blot analyses, following the previously described protocol [42]. The protein content was quantified using DC^TM^ Protein assay reagents sourced from Bio-Rad Laboratories (Hercules, CA, USA). The Western blot analysis utilized the following primary antibodies: rabbit anti-Nrf2 (cat. no. sc-722), rabbit anti-NF-κB (cat. no. sc-8414), and goat anti-β-actin (cat. no. sc-1616) antibodies, acquired from Santa Cruz Biotechnology Inc. (Dallas, TX, USA). Mouse anti-Cu/Zn-SOD (cat. No. 556360) and anti-Mn-SOD (cat. No. 611580) antibodies were obtained from BD Biosciences (San Jose, CA, USA). Rabbit anti-glutathione peroxidase 1 (GPx1) (cat. no. ab22604) was purchased from Abcam plc. (Cambridge, UK). All primary antibodies diluted at 1/1000 were incubated for 2 h at RT. Horseradish peroxidase-labeled secondary anti-mouse, goat, or rabbit IgG antibodies were procured from Santa Cruz Biotechnology Inc. (Dallas, TX, USA). All secondary antibodies diluted at 1/10,000 were incubated for 1 h at RT. Proteins were visualized using chemiluminescence detection (PerkinElmer Life Sciences, Inc., Boston, WA, USA). β-actin was utilized as the internal control, and each targeted band was normalized to the corresponding β-actin band. Subsequently, a quantitative analysis of the study group data was performed relative to the control group to assess the observed changes. Each experiment was carried out in triplicate for accuracy and consistency.

### 4.8. Real-Time Quantitative PCR Analysis (RT-qPCR) 

SW1353 cells were seeded at a density of 4 × 10^5^ cells in 6 cm dishes. The cells were then treated with MIA and/or Se for 24 h. After treatment, total RNA was extracted from the cells using TRIzol^®^ reagent from Thermo Fisher Scientific Inc. (Waltham, MA, USA) as per the manufacturer’s instructions. From the total RNA, cDNA was synthesized using 250 ng of random primers, 200 μM of dNTPs, 200 units of recombinant RNasin ribonuclease inhibitor, and 200 units of M-MLV reverse transcriptase (Promega Corporation, Madison, WI, USA). The cDNA was used for real-time quantitative PCR, which was performed on a MiniOpticon™ Real-Time PCR Detection System (Bio-Rad Laboratories, Hercules, CA, USA) following the manufacturer’s protocol. The PCR amplification reaction mixture (20 μL) contained 100 ng of cDNA, 10 μL of iQ™ SYBR^®^ Green Supermix (Bio-Rad Laboratories, Hercules, CA, USA), and 200 nM of gene-specific primer pairs for GCLC, GCLM, GSS, GR, GPx1, MMP-1, -3, -9, -13, ADAMTS-4, IL-1β, TNF-α, IL-6, IL-17A, or GAPDH. The PCR products from each primer pair were subjected to melting curve analysis to confirm amplification specificity. The reaction conditions were initial denaturation at 95 °C for 10 min, followed by 40 cycles of denaturation at 95 °C for 15 s, and annealing at 60 °C for 60 s. After RT-qPCR, the temperature was increased from 70 to 95 °C at a rate of 0.2 °C per second to construct a melting curve. The primers and amplified products for each gene used in the study are detailed in Table 1. All primers were designed and confirmed for their specificity for the target gene using the NCBI Primer-BLAST tool. The RT-qPCR experiments adhered to the Minimum Information for Publication of Quantitative Real-Time PCR Experiments (MIQE) guidelines [43]. The cycle threshold (Ct) value of the target gene was normalized to GAPDH. Data were calculated and expressed as 2^−ΔΔCt^ [44] using MJ Opticon Monitor Analysis software version 3.1 (Bio-Rad Laboratories, Hercules, CA, USA). The 2^−∆∆Ct^ method presupposes amplification efficiencies close to 100% ± 5% of the target and reference genes across all samples [45]. In this study, the efficiency ranged from 97.5% to 101.9%.

### 4.9. Animals and Treatments

The 4-week-old male Wistar rats were procured from BioLASCO Taiwan Co., Ltd. (Charles River Technology, Taipei, Taiwan). After environmental accommodation for one week, twenty-four male Wistar rats were randomly divided into four groups, each comprising 6 rats. The four groups were as follows: control without treatment (C group), control with Se supplementation (Se group), rats injected with MIA (MIA group), and rats injected with MIA and receiving Se supplementation (MIA+Se group). Anesthesia was induced using a Tiletamine and Zolazepam mixture (Zoletil 50) (Virbac, Carros, France), and a single intra-articular injection was administered either with sterile saline or MIA (Sigma-Aldrich, St. Louis, MO, USA). The MIA injection was a dose of 3 mg of MIA in 20 μL of 0.9% sterile saline, delivered through the infrapatellar ligament of the left knee using a 31-gauge needle. Control animals received a single intra-articular injection of 20 μL of 0.9% sterile saline into the left knee. Following the single MIA injection to simulate the progression of osteoarthritis (OA), all groups of rats were provided with standard rodent chow containing 0.25 ppm of Se (Altromin, Lage, Germany) for an additional two weeks. Simultaneously, the Se supplementation groups received an additional Se dosage of 20.7 μg of Se/kg/day dissolved in water via gavage. After the experimental period, rats were euthanized using CO_2_, and the left legs were extracted for histomorphometric analyses. All collected samples were stored at −80 °C until further analysis. The study adhered to the Guide for the Care and Use of Laboratory Animals of the United States National Institutes of Health. The animal use protocol was reviewed and approved by the Institutional Animal Care and Use Committee (IACUC) of Kaohsiung Medical University (Approval No. 103200).

### 4.10. Histopathology of Joint Tissues in Rats

The histopathology of the rat’s left joints was examined using safranin O and fast green stains (Sigma-Aldrich, St. Louis, MO, USA), following the procedure detailed in a previous study [18]. Safranin O is capable of staining cartilage tissue in red, while fast green specifically stains tissues other than cartilage in green. This staining procedure facilitates the observation of acidic proteoglycan in the cartilage. Following the safranin O and fast green staining, the severity of joint damage in the cartilage tissue was assessed using the Osteoarthritis Research Society International (OARSI) scoring criteria. In this grading system, the degree of osteoarthritis (OA) cartilage matrix loss is delineated by grades ranging from 0 (normal) to 6 (severe), with higher scores indicative of more significant damage to the cartilage tissue [46]. This scoring system was employed to assess the severity of cartilage degeneration in the knee joints.

### 4.11. Measurement of Serum Biomarkers in Rats

After sacrificing the rats, serum was obtained by centrifuging the blood samples at 3000× *g* for 15 min. The collected serum samples were then divided into aliquots and stored at −80 °C until further use. To maintain sample integrity, no repeated freezing and thawing of specimens occurred prior to measurements. Inflammatory markers, including IL-1β (cat. no. EL0040Ra), TNF-α (cat. no. SL0722Ra), and IL-6 (cat. no. SL0411Ra), were quantified using rat-specific ELISA kits (Sunlong Biotech Co., Ltd., Hangzhou, Zhejiang, China). Similarly, the extracellular matrix-degrading enzyme MMP-13 was assessed using rat MMP-13 ELISA kits (cat. no. SL0482Ra) (Sunlong Biotech Co., Ltd., Hangzhou, Zhejiang, China). SOD and GSH levels were determined using a SOD assay kit (Item No. 706002) and a GSH assay kit (Item No. 703002) (Cayman Chemical, Ann Arbor, MI, USA), respectively. All the samples were examined in triplicate within each assay to ensure the accuracy and reliability of the results.

### 4.12. Statistical Analysis

The data are expressed as means ± standard deviations (S.D.s). Statistical differences between the control and treated groups were assessed using an ANOVA followed by a Fisher’s Exact Test. All statistical analyses were conducted using SAS version 6.011 software (SAS Institute Inc., Cary, NC, USA). A *p*-value less than 0.05 was considered statistically significant.

## 5. Conclusions

The present study demonstrated that exposure to MIA leads to increased levels of ROS, pro-inflammatory cytokines, and MMPs in chondrocytes (SW1353 cells). However, this harmful effect can be prevented through treatment with Se. This can protect chondrocytes from MIA-induced harm and cartilage loss by enhancing their antioxidant capacity through the Nrf2 pathway and inhibiting inflammation via the NF-κB pathway. These findings suggest that Se supplementation could be beneficial in the treatment of OA progression in humans.

## Figures and Tables

**Table 1:** Primer sets for RT-qPCR analysis.

Primer Name	NCBI Reference Sequence	Primer Sequence (5′->3′)	Amplicon Length (bp)
GAPDH	NM_002046.5	F: ACAGTCAGCCGCATCTTC R: GCCCAATACGACCAAATCC	101
GCLC	NM_001498	F: GAGGTCAAACCCAACCCAGT R: AAGGTACTGAAGCGAGGGTG	92
GCLM	XM_005270754.3	F: CTTGGAGCATTTACAGCCTTAC R: GGTGGCATCACACAGCAG	177
GSS	NM_000178	F: GCGGAGGAAAGGCGAACTA R: AGAGCGTGAATGGGGCATAG	184
GR	NM_000637.3	F: TGAAGTTCTCCCAGGTCAAGG R: TCAGGTCCTTGGTATTCGGGA	159
GPx1	NM_000581.2	F: GAATGTGGCGTCCCTCTG R: TCGTTCTTGGCGTTCTCC	141
MMP-1	NM_002421.3	F: AGATGTGGAGTGCCTGATGTG R: CTTGACCCTCAGAGACCTTGG	199
MMP-3	NM_002422.3	F: CCACTCTATCACTCACTCACAG R: GACAGCATCAAAGGACAAAGC	186
MMP-9	NM_004994.2	F: CTGGTCCTGGTGCTCCTG R: TGCCTGTCGGTGAGATTGG	110
MMP-13	NM_002427.3	F: GACCCTGGAGCACTCATGTTTC R: TCCTCGGAGACTGGTAATGGC	192
ADAMTS-4	NM_005099.4	F: AGGCAGTGATGGGTTAGTGG R: CCTAGTCCTTGTCCCCTTCC	178
IL-1β	NM_000576.2	F: TGATGGCTTATTACAGTGGCAATG R: GTAGTGGTGGTCGGAGATTCG	140
TNF-α	NM_000594.3	F: TCAGCAAGGACAGCAGAGGAC R: GGAGCCGTGGGTCAGTATGTG	138
IL-6	NM_000600.4	F: ACCCCCAATAAATATAGGACTGGA R: GAGAAGGCAACTGGACCGAA	145
IL-17A	NM_002190.2	F: GGCTGGAGAAGATACTGGTGTC R: AGGCTGTCTTTGAAGGATGAGG	158

GAPDH: glyceraldehyde-3-phosphate dehydrogenase; GCLC: glutamate–cysteine ligase catalytic subunit; GCLM: glutamate–cysteine ligase modifier subunit; GSS: glutathione synthetase; GPx1: glutathione peroxidase 1; GR: glutathione reductase; MMP: matrix metalloproteinase; ADAMTS-4: a disintegrin and metalloproteinase with thrombospondin motifs-4; IL: interleukin; TNF-α: tumor necrosis factor alpha.

## Data Availability

Data are contained within the article and supplementary materials.

## Supplementary Materials

The following supporting information can be downloaded at https://www.mdpi.com/article/10.3390/ijms25052511/s1.
