# Supplementary material for: Selenium Lessens Osteoarthritis by Protecting Articular Chondrocytes from Oxidative Damage through Nrf2 and NF-κB Pathways
Source: Int J Mol Sci. 2024 Feb 21;25(5):2511. doi: 10.3390/ijms25052511 (PMC10931631; doi:10.3390/ijms25052511)

Supplementary Figure S1

The images of cartilage morphology and histology in MIA-induced OA rats with or without Se supplementation.

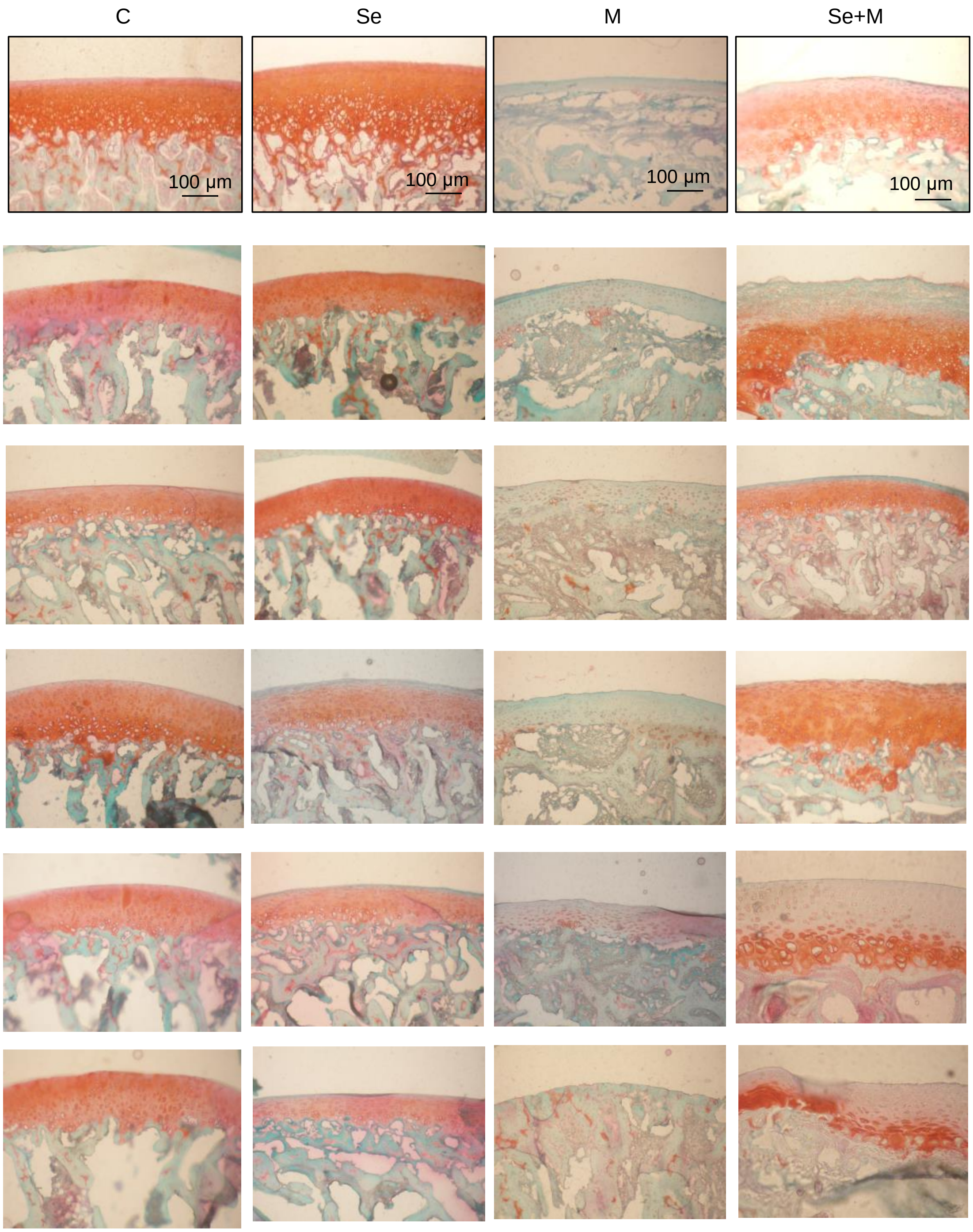

Supplement: Supplementary file 1 [file ijms-25-02511-s001.zip › ijms-2871206-supplementary.pdf]
